# Effect of pleural invasion on survival of patients with small cell lung cancer: Propensity score analysis and nomogram establishment based on the SEER database

**DOI:** 10.3389/fsurg.2023.1108732

**Published:** 2023-02-23

**Authors:** Jie Yang, Hui Yin, Mingshan Liu, Guowen Zou, Bentong Yu

**Affiliations:** ^1^ Department of Thoracic Surgery, The First Affiliated Hospital of Nanchang University, Nanchang, China; ^2^ Department of Thoracic Surgery, The First Affiliated Hospital of Shaoyang University, Shaoyang, China

**Keywords:** small cell lung cancer, pleural invasion, overall survival, prognostic factor, nomograms

## Abstract

**Objectives:**

Pleural invasion (PI) is identified as an adverse prognostic factor for non-small cell lung cancer (NSCLC), but its value in small cell lung cancer (SCLC) remains unclear. We aimed to evaluate the survival effect of PI on overall survival (OS) in SCLC, meanwhile, we established a predictive nomogram based on related risk factors for OS in SCLC patients with PI.

**Methods:**

We extracted the data of patients diagnosed with primary SCLC between 2010 and 2018 from the Surveillance, Epidemiology, and End Results (SEER) database. The propensity score matching (PSM) method was used to minimize the baseline difference between the non-PI and PI groups. Kaplan-Meier curves and the log-rank test were used for survival analysis. Univariate and multivariate Cox regression analyses were applied to identify the independent prognostic factors. Randomly divided the patients with PI into training (70%) and validation (30%) cohorts. A prognostic nomogram was established based on the training cohort and was evaluated in the validation cohort. The C-index, receiver operating characteristic curves (ROC), calibration curves, and decision curve analysis (DCA) were applied to assess the performance of the nomogram.

**Results:**

A total of 1,770 primary SCLC patients were enrolled, including1321patients with non-PI and 449 patients with PI. After PSM, the 387 patients in the PI group matched the 387 patients in the non-PI group. By Kaplan-Meier survival analysis, we observed the exact beneficial effect of non-PI on OS in both original and matched cohorts. Multivariate Cox analysis showed similar results to demonstrate a statistically significant benefit for patients with non-PI in both original and matched cohorts. Age, N stage, M stage, surgery, radiotherapy, and chemotherapy were independent prognostic factors for SCLC patients with PI. The C-index of the nomogram in the training and validation cohort was 0.714 and 0.746, respectively. The ROC curves, calibration curves, and DCA curves also demonstrated good predictive performance in the training and validation cohorts of the prognostic nomogram.

**Conclusion:**

Our study shows that PI is an independent poor prognostic factor for SCLC patients. The nomogram is a useful and reliable tool to predict the OS in SCLC patients with PI. The nomogram can provide strong references to clinicians to facilitate clinic decisions.

## Introduction

According to the Global Cancer Statics 2020, lung cancer is the leading cause of cancer mortality worldwide, with an estimated 2.2 million new cases and 1.8 million new deaths in 2020 (1). SCLC is characterized as one of the most lethal and aggressive types which accounts for around 15% of lung cancer, while NSCLC accounts for nearly 80%. It is widely known that SCLC is a recalcitrant carcinoma with remarkable metastatic and recurrent proclivity. As previous studies have reported, only one-third of SCLC patients are initially diagnosed with early-stage, while close to 70% of the cases are advanced stage at diagnosis (2, 3). In a comprehensive analysis of 358 SCLC patients with extensive-stage, 43.8% of patients died within 12 months (4). Another study from China also reported that the median OS was just 11 months and 58.4% of patients with extensive-stage SCLC died within 1 year (5). To sum up, patients with SCLC have a worse prognosis which would be influenced by many factors such as age, lymph node metastasis, distant metastasis, and so on. Nonetheless, the influence of pleural invasion on prognosis remains unknown.

Pleural invasion (PI) has been established as a negative prognostic factor in NSCLC and was first included as a non-size based T2 descriptor in the 5th edition AJCC staging system in 1997 (6). Pathological PI is classified into the following subgroups according to the International Association for the Study of Lung Cancer: PL0, the tumor grows within the parenchyma or does not completely penetrate the elastic layer; PL1, the tumor extends beyond the elastic layer; PL2, tumor invades into the surface of the visceral pleura; PL3, tumor invades into or through the parietal pleura (7, 8). Generally speaking, PL0 indicates no evidence of PI, while PL1, PL2, and PL3 stand for tumor invasion of pleura. In the 8th TNM staging system, tumors ≤ 3 cm (T1a or T1b) with PL1 or PL2 are upgraded to T2a while tumors with PL3 are defined as T3. Previous research has reported that NSCLC patients with visceral pleural invasion (VPI) are associated with a higher incidence of malignant pleural effusion, mediastinal lymph node metastasis, and postoperative recurrence (9–11). In a retrospective study of 2,657 patients with T1-4N0-2M0 NSCLC, they demonstrated that VPI was the strongest significant independent predictor of recurrence in patients with pathological stage I treated without adjuvant chemotherapy (12). In another series of 16,315 NSCLC patients with stage I–II, VPI occurred in 3,389 patients (21%) and it was a prevalent finding associated with worse prognosis, even among patients with tumors > 3 cm (13). Although there were some studies investigating prognostic factors for SCLC, most of them ignored the effect of PI on survival or were limited to a small number of cases (14–17). However, the effect of PI on OS in patients with SCLC is seldom reported.

As a widely used method, nomograms can accurately predict OS of cancer patients. Most existing nomograms, however, are derived from NSCLC patients with VPI. Nomograms for the survival of SCLC patients with PI have not been published until now.

Therefore, we aimed to determine whether the presence of PI could influence OS in SCLC patients and construct a novel nomogram to predict OS in SCLC patients with PI based on the demographic and clinicopathologic variables from the SEER database. As a result, this could facilitate individualized patient care as well as medical therapy.

## Materials and methods

### Data source

We used the specialized database “Incidence–SEER Research Plus Data, 18 Registries, Nov 2020 Sub (2000–2018)” to extract data using the SEER*Stat software, version 8.4.0. For available publicly and access to the SEER database as well as without individual information of patients, informed consent was not required in the present study.

### Patients collection

**Figure 1:**
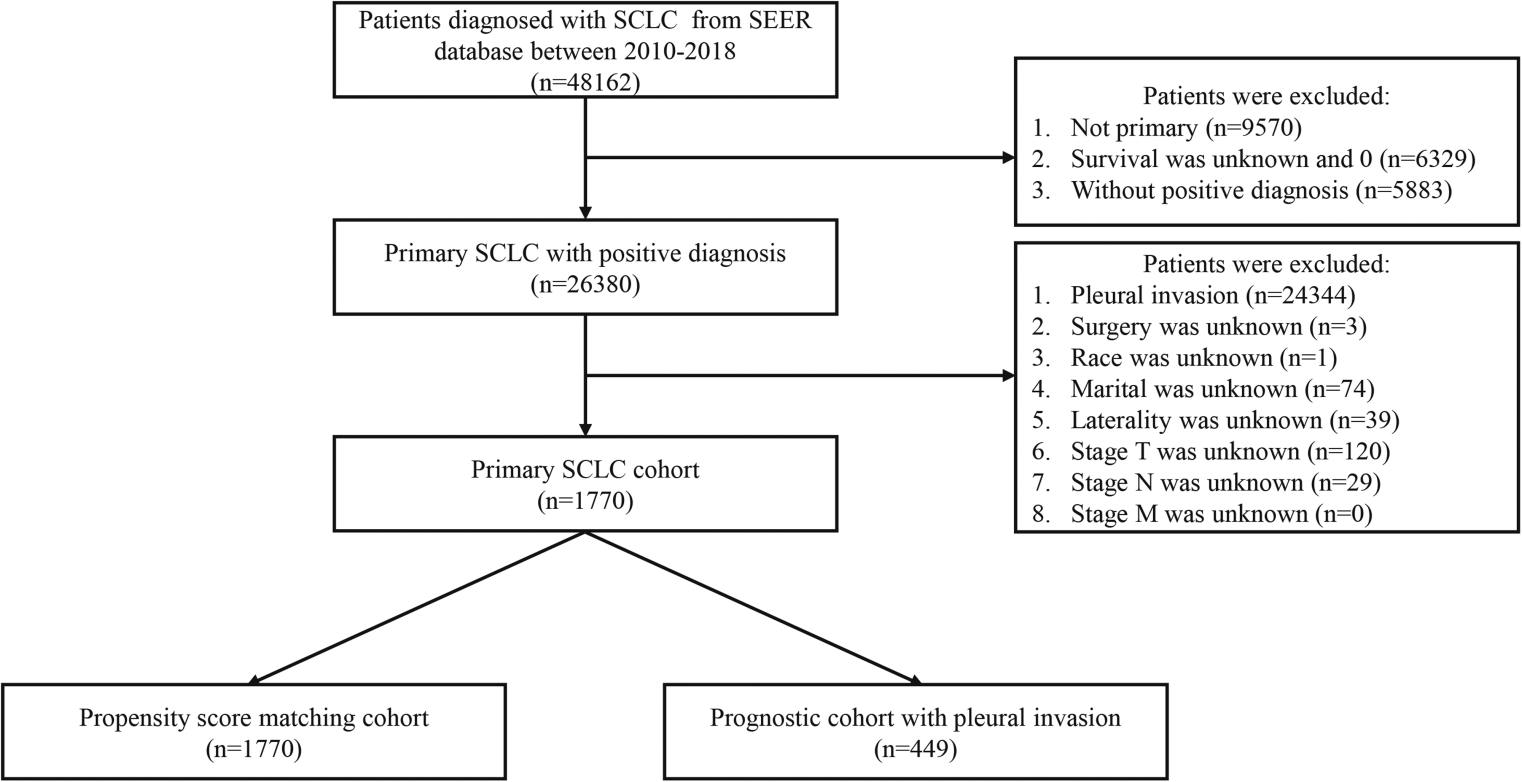
The selection process of the present study. SCLC: small cell lung cancer; PI: pleural invasion.

Because the PI status has been recorded since 2010 based on the term, cs site-specific factor 2, patients were identified from the SEER database between 2010 and 2018. The inclusion criteria were as follows: (a) malignant tumor located in the main bronchus and lung (Site code: C340-C349); (b) patients diagnosed with primary SCLC (Histology code: 8002, 8041, 8042, 8043, 8044, 8045); (c) diagnostic confirmation based on positive histology or positive microscopic confirmation; (d) the status of PI was recorded clearly; (e) T stage, N stage, and M stage according to the 7th edition AJCC staging system was complete. Besides, clinical variables including age at diagnosis, sex, race, grade, tumor site, laterality, surgery, radiotherapy, and chemotherapy were contained. The exclusion criteria were as follows: (a) survived less than 1 month after diagnosis; (b) patients aged < 18 years; (c) unknown data on race, marital, laterality, surgery, radiotherapy, and chemotherapy. Finally, a total of 1,770 patients met the criteria in the original cohort and 449 patients in the prognostic cohort. Age at diagnosis was divided into under 60 years old, 60–70 years old, and over 70 years old. Tumor grade was grouped into grade I–II, grade III, grade IV, and unknown. All the selected processes of the two study cohorts were exhibited in Figure 1.

### Statistical analysis

In the present study, R software (version 4.0.3) was applied to perform all statistical analyses, and *P*-value < 0.05 (two sides) was considered statistical significance. In this study, we transformed all continuous variables into categorical variables except survival time to simplify the analyses. Count and percentage were used to summarize categorical variables.

In the original cohort, the distribution and difference between the PI and non-PI groups were examined by the Chi-square test or Fisher's exact test. To eliminate baseline differences in the two groups, the propensity score matching (PSM) method was applied using the “MatchIt” package in R software (Match Ratio 1:1; Logit model; the nearest neighbor matching approach). OS was defined as the time between confirming SCLC to any cause of death. To compare OS status between patients with the PI and non-PI groups, Kaplan-Meier survival curves were generated by the log-rank test before and after PSM. To evaluate the impact of PI on OS, the univariate and multivariate Cox proportional hazards regression analyses were adopted to confirm independent OS-related factors.

In the prognostic cohort with PI, training and validation cohorts were extracted by R software with a ratio of 7:3 randomly, meanwhile, the distribution and difference between the two cohorts were examined by the Chi-square test or Fisher's exact test. The univariate and multivariate Cox proportional hazards regression analyses were performed to determine independent OS-related factors. Risk factors with *P*-value < 0.05 in the univariate analysis were further analyzed in multivariate analysis. A prognostic nomogram was constructed based on the independent prognostic factors in the training cohort and was validated in the validation cohort by the “rms” package. Time-dependent receiver operating characteristic (ROC) curves were performed to predict 1-year, 2-year, and 3-year overall survival, and the corresponding area under the curve (AUC) was calculated to show the discrimination as well as the C-index. To determine the consistency between predicted and actual probability, calibration curves were plotted. Decision curve analysis (DCA) curves were generated to evaluate the clinical benefits and improved performance of the nomogram.

## Results

### Baseline characteristics in PSM cohort

After screening patients based on the specific inclusion and exclusion criteria, 1,770 patients were enrolled in our study. The median follow-up time was 12 months and 1,405 (79.4%) deaths were observed. Finally, 1,321 patients (74.6%) were assigned to the non-PI group and 449 patients (25.4%) were assigned to the PI group. Table 1 summarized the baseline characteristics of the two groups. Significant differences in histology, laterality, tumor site, T stage, M stage, surgery, and radiotherapy were observed between the two cohorts.

**Table 1:** Baseline characteristics between PI and non-PI groups among patients with SCLC in the original cohort and matched cohort.

	Originl cohort				Matched cohort			
	All	Non-PI	PI	*P*	All	Non-PI	PI	*P*
	(*N* = 1770)	(*N* = 1321)	(*N* = 449)		(*N* = 774)	(*N* = 387)	(*N* = 387)	
**Age**								
<60	399 (22.5%)	314 (23.8%)	85 (18.9%)	0.102	155 (20.0%)	75 (19.4%)	80 (20.7%)	0.539
60–70	725 (41.0%)	532 (40.3%)	193 (43.0%)		338 (43.7%)	164 (42.4%)	174 (45.0%)	
>70	646 (36.5%)	475 (36.0%)	171 (38.1%)		281 (36.3%)	148 (38.2%)	133 (34.4%)	
**Sex**								
Female	901 (50.9%)	685 (51.9%)	216 (48.1%)	0.172	377 (48.7%)	188 (48.6%)	189 (48.8%)	1.000
Male	869 (49.1%)	636 (48.1%)	233 (51.9%)		397 (51.3%)	199 (51.4%)	198 (51.2%)	
**Race**								
Black	171 (9.7%)	123 (9.3%)	48 (10.7%)	0.137	85 (11.0%)	41 (10.6%)	44 (11.4%)	0.529
Other	98 (5.5%)	66 (5.0%)	32 (7.1%)		47 (6.1%)	20 (5.2%)	27 (7.0%)	
White	1,501 (84.8%)	1,132 (85.7%)	369 (82.2%)		642 (82.9%)	326 (84.2%)	316 (81.7%)	
**Marital**								
Married	948 (53.6%)	701 (53.1%)	247 (55.0%)	0.560	422 (54.5%)	214 (55.3%)	208 (53.7%)	0.634
Other	552 (31.2%)	421 (31.9%)	131 (29.2%)		228 (29.5%)	116 (30.0%)	112 (28.9%)	
Single	270 (15.3%)	199 (15.1%)	71 (15.8%)		124 (16.0%)	57 (14.7%)	67 (17.3%)	
**Grade**								
I–II	35 (2.0%)	29 (2.2%)	6 (1.3%)	0.093	11 (1.4%)	5 (1.3%)	6 (1.6%)	0.948
III	342 (19.3%)	239 (18.1%)	103 (22.9%)		150 (19.4%)	73 (18.9%)	77 (19.9%)	
IV	371 (21.0%)	275 (20.8%)	96 (21.4%)		165 (21.3%)	85 (22.0%)	80 (20.7%)	
Unknown	1,022 (57.7%)	778 (58.9%)	244 (54.3%)		448 (57.9%)	224 (57.9%)	224 (57.9%)	
**Histology**								
8041	1,569 (88.6%)	1,182 (89.5%)	387 (86.2%)	0.013	683 (88.2%)	341 (88.1%)	342 (88.4%)	0.802
8042	40 (2.3%)	34 (2.6%)	6 (1.3%)		10 (1.3%)	4 (1.0%)	6 (1.6%)	
8044	5 (0.3%)	3 (0.2%)	2 (0.4%)		3 (0.4%)	1 (0.3%)	2 (0.5%)	
8045	156 (8.8%)	102 (7.7%)	54 (12.0%)		78 (10.1%)	41 (10.6%)	37 (9.6%)	
**Laterality**								
Bilateral	13 (0.7%)	7 (0.5%)	6 (1.3%)	0.023	8 (1.0%)	4 (1.0%)	4 (1.0%)	0.880
Left	742 (41.9%)	536 (40.6%)	206 (45.9%)		353 (45.6%)	181 (46.8%)	172 (44.4%)	
Right	1,015 (57.3%)	778 (58.9%)	237 (52.8%)		413 (53.4%)	202 (52.2%)	211 (54.5%)	
**Tumor site**								
Main bronchus	137 (7.7%)	113 (8.6%)	24 (5.3%)	<0.001	44 (5.7%)	20 (5.2%)	24 (6.2%)	0.993
Upper lobe	949 (53.6%)	720 (54.5%)	229 (51.0%)		392 (50.6%)	197 (50.9%)	195 (50.4%)	
Middle lobe	90 (5.1%)	76 (5.8%)	14 (3.1%)		28 (3.6%)	14 (3.6%)	14 (3.6%)	
Lower lobe	418 (23.6%)	305 (23.1%)	113 (25.2%)		188 (24.3%)	95 (24.5%)	93 (24.0%)	
Overlapping lesion	21 (1.2%)	11 (0.8%)	10 (2.2%)		15 (1.9%)	7 (1.8%)	8 (2.1%)	
Lung, NOS	155 (8.8%)	96 (7.3%)	59 (13.1%)		107 (13.8%)	54 (14.0%)	53 (13.7%)	
**T**								
T1	428 (24.2%)	428 (32.4%)	0 (0%)	<0.001	0	0	0	0.673
T2	492 (27.8%)	318 (24.1%)	174 (38.8%)		277 (35.8%)	135 (34.9%)	142 (36.7%)	
T3	362 (20.5%)	232 (17.6%)	130 (29.0%)		219 (28.3%)	107 (27.6%)	112 (28.9%)	
T4	488 (27.6%)	343 (26.0%)	145 (32.3%)		278 (35.9%)	145 (37.5%)	133 (34.4%)	
**N**								
N0	581 (32.8%)	448 (33.9%)	133 (29.6%)	0.360	199 (25.7%)	97 (25.1%)	102 (26.4%)	0.709
N1	211 (11.9%)	157 (11.9%)	54 (12.0%)		89 (11.5%)	42 (10.9%)	47 (12.1%)	
N2	777 (43.9%)	566 (42.8%)	211 (47.0%)		384 (49.6%)	192 (49.6%)	192 (49.6%)	
N3	201 (11.4%)	150 (11.4%)	51 (11.4%)		102 (13.2%)	56 (14.5%)	46 (11.9%)	
**M**								
M0	975 (55.1%)	773 (58.5%)	202 (45.0%)	<0.001	322 (41.6%)	155 (40.1%)	167 (43.2%)	0.422
M1	795 (44.9%)	548 (41.5%)	247 (55.0%)		452 (58.4%)	232 (59.9%)	220 (56.8%)	
**Surgery**								
No	1,130 (63.8%)	870 (65.9%)	260 (57.9%)	0.003	511 (66.0%)	260 (67.2%)	251 (64.9%)	0.544
Yes	640 (36.2%)	451 (34.1%)	189 (42.1%)		263 (34.0%)	127 (32.8%)	136 (35.1%)	
**Radiation**								
No	926 (52.3%)	657 (49.7%)	269 (59.9%)	<0.001	435 (56.2%)	216 (55.8%)	219 (56.6%)	0.885
Yes	844 (47.7%)	664 (50.3%)	180 (40.1%)		339 (43.8%)	171 (44.2%)	168 (43.4%)	
**Chemotherapy**								
No	397 (22.4%)	301 (22.8%)	96 (21.4%)	0.556	165 (21.3%)	84 (21.7%)	81 (20.9%)	0.861
Yes	1,373 (77.6%)	1,020 (77.2%)	353 (78.6%)		609 (78.7%)	303 (78.3%)	306 (79.1%)	

8041: small cell carcinoma, NOS; 8042: oat cell carcinoma; 8043: small cell carcinoma, intermediate cell; 8045: combined small cell carcinoma; PI: pleural invasion.

### Survival analysis between non-Pi and Pi groups

**Figure 2:**
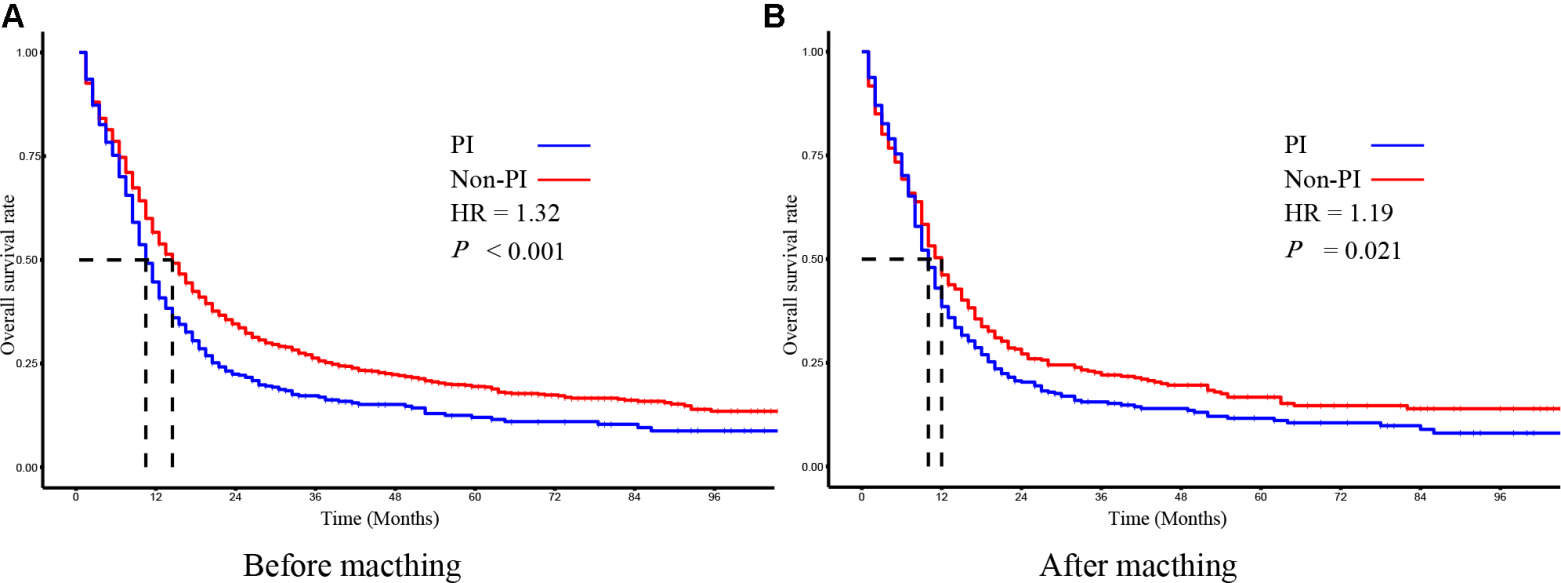
The kaplan–meier survival analysis of SCLC patients with PI and without PI before (**A**) and after (**B**) matching.

In the non-PI group, the 1-year OS rate, 2-year OS rate, and 3-year OS rate were 53.9%, 34.8%, and 28.3%, respectively. While in the PI group, the 1-year OS rate, 2-year OS rate, and 3-year OS rate were 41.2%, 23.6%, and 19.4%, respectively. The median OS time of the non-PI and PI groups was 14 months and 10 months, respectively. As determined by Kaplan-Meier analysis, patients with PI had a significantly lower overall survival than patients without PI (Figure 2A).

To evaluate the effect of PI on survival, we applied the PSM method to diminish the measurable confounders between the two groups. As summarized in Table 1, both non-PI and PI groups comprised 387 patients with similar baseline characteristics for further analysis. The median OS time of the non-PI and PI groups was 12 months and 10 months, respectively. Kaplan-Meier plots of OS also revealed that patients with PI had significantly worse OS than patients without PI (Figure 2B).

To adjust potential modifier effects to PI, multivariate Cox regression analysis in the original and the propensity score-matched cohorts was fitted for overall survival. As shown in Tables 2, 3, PI indeed brought a negative effect on overall survival in both original and matched cohorts. Additionally, surgery, radiotherapy, and chemotherapy could improve OS in these two cohorts. In both cohorts, advanced age, located in the overlapping lesion of the lung, lymph node metastasis, and distant metastasis were associated with poor prognosis. Higher grade and higher T stage were independent risk factors for OS in the original cohort whereas they did not influence OS in the matched cohort.

**Table 2:** Univariate and multivariate Cox regression analyses of prognostic factors associated with OS in SCLC patients in the original cohort.

	Univariate analysis			Multivariate analysis		
	HR	95%CI	*P*	HR	95%CI	*P*
**Age, years**						
<60	Reference					
60–70	1.178	1.024–1.356	0.022	1.166	1.011–1.345	0.035
>70	1.550	1.345–1.786	<0.001	1.647	1.425–1.905	<0.001
**Sex**						
Female	Reference					
Male	1.208	1.088–1.342	<0.001	1.105	0.993–1.229	0.066
**Race**						
Black	Reference					
Other	1.088	0.824–1.435	0.552			
White	0.941	0.786–1.128	0.513			
**Marital**						
Married	Reference					
Other	1.081	0.961–1.216	0.194			
Single	1.140	0.981–1.326	0.088			
**Grade**						
I–II	Reference					
III	1.882	1.133–3.126	0.015	1.771	1.058–2.966	0.030
IV	2.518	1.521–4.169	<0.001	2.049	1.224–3.430	0.006
Unknown	3.641	2.220–5.974	<0.001	1.984	1.189–3.311	0.009
**Histology**						
8041	Reference					
8042	1.398	1.008–1.939	0.045	1.030	0.736–1.441	0.864
8044	0.697	0.261–1.861	0.472	0.552	0.204–1.493	0.242
8045	0.556	0.452–0.684	<0.001	1.060	0.847–1.328	0.609
**Laterality**						
Bilateral	Reference					
Left	0.490	0.283–0.849	0.011	1.229	0.694–2.177	0.479
Right	0.512	0.296–0.886	0.017	1.250	0.708–2.208	0.441
**Site**						
Main bronchus	Reference					
Upper lobe	0.587	0.485–0.710	<0.001			
Middle lobe	0.603	0.446–0.815	0.001			
Lower lobe	0.629	0.512–0.774	<0.001			
Overlapping lesion	1.086	0.662–1.780	0.744			
Lung, NOS	1.150	0.905–1.461	0.253			
**T**						
T1	Reference					
T2	1.651	1.408–1.937	<0.001	1.168	0.987–1.382	0.071
T3	2.590	2.194–3.057	<0.001	1.386	1.157–1.661	<0.001
T4	3.158	2.702–3.692	<0.001	1.418	1.183–1.701	<0.001
**N**						
N0	Reference					
N1	1.595	1.326–1.919	<0.001	1.423	1.178–1.718	<0.001
N2	2.516	2.210–2.864	<0.001	1.618	1.393–1.879	<0.001
N3	3.265	2.729–3.905	<0.001	1.714	1.405–2.091	<0.001
**M**						
M0	Reference					
M1	3.490	3.127–3.896	<0.001	2.167	1.904–2.466	<0.001
**Surgery**						
No	Reference					
Yes	0.298	0.264–0.336	<0.001	0.526	0.444–0.621	<0.001
**Radiotherapy**						
No	Reference					
Yes	0.894	0.805–0.992	0.035	0.834	0.741–0.939	0.003
**Chemotherapy**						
No	Reference					
Yes	0.810	0.714–0.919	0.001	0.593	0.516–0.681	<0.001

8041: small cell carcinoma, NOS; 8042: oat cell carcinoma; 8043: small cell carcinoma, intermediate cell; 8045: combined small cell carcinoma.

**Table 3:** Univariate and multivariate Cox regression analyses of prognostic factors associated with OS in SCLC patients in the matched cohort.

	Univariate analysis			Multivariate analysis		
	HR	95%CI	*P*	HR	95%CI	*P*
**Age, years**						
<60	Reference					
60–70	1.285	1.038–1.591	0.021	1.137	0.913–1.418	0.253
>70	1.649	1.326–2.052	<0.001	1.425	1.135–1.791	0.002
**Sex**						
Female	Reference					
Male	1.143	0.980–1.334	0.090			
**Race**						
Black	Reference					
Other	0.966	0.654–1.427	0.863			
White	0.894	0.699–1.144	0.372			
**Marital**						
Married	Reference					
Other	1.136	0.953–1.354	0.156			
Single	1.016	0.816–1.266	0.884			
**Grade**						
I–II	Reference					
III	1.443	0.671–3.103	0.348	1.482	0.668–3.291	0.333
IV	1.761	0.822–3.769	0.145	1.568	0.707–3.479	0.268
Unknown	2.912	1.378–6.152	0.005	2.003	0.905–4.433	0.086
**Histology**						
8041	Reference					
8042	1.300	0.695–2.431	0.411	1.059	0.560–2.003	0.859
8044	0.452	0.113–1.810	0.262	0.318	0.077–1.309	0.113
8045	0.550	0.415–0.729	<0.001	1.103	0.809–1.503	0.536
**Laterality**						
Bilateral	Reference					
Left	0.616	0.305–1.243	0.176			
Right	0.585	0.290–1.179	0.134			
**Site**						
Main bronchus	Reference					
Upper lobe	0.625	0.449–0.870	0.005	1.021	0.726–1.434	0.907
Middle lobe	0.560	0.327–0.959	0.035	1.082	0.628–1.864	0.777
Lower lobe	0.679	0.480–0.961	0.029	1.076	0.751–1.541	0.690
Overlapping lesion	1.480	0.805–2.722	0.207	2.313	1.238–4.322	0.009
Lung, NOS	1.103	0.765–1.592	0.599	1.096	0.750–1.603	0.635
**T**						
T2	Reference					
T3	1.741	1.425–2.125	<0.001	0.958	0.771–1.190	0.695
T4	2.102	1.742–2.536	<0.001	0.963	0.770–1.204	0.740
**N**						
N0	Reference					
N1	1.515	1.136–2.022	0.005	1.754	1.299–2.368	<0.001
N2	2.471	2.011–3.037	<0.001	1.817	1.431–2.307	<0.001
N3	3.005	2.298–3.930	<0.001	1.601	1.183–2.167	0.002
**M**						
M0	Reference					
M1	3.453	2.910–4.099	<0.001	2.448	1.963–3.053	<0.001
**Surgery**						
No	Reference					
Yes	0.295	0.246–0.354	<0.001	0.578	0.443–0.753	<0.001
**Radiotherapy**						
No	Reference					
Yes	0.791	0.677–0.924	0.003	0.851	0.718–1.008	0.061
**Chemotherapy**						
No	Reference					
Yes	0.763	0.631–0.923	0.005	0.460	0.370–0.570	<0.001

8041: small cell carcinoma, NOS; 8042: oat cell carcinoma; 8043: small cell carcinoma, intermediate cell; 8045: combined small cell carcinoma.

**Figure 3:**
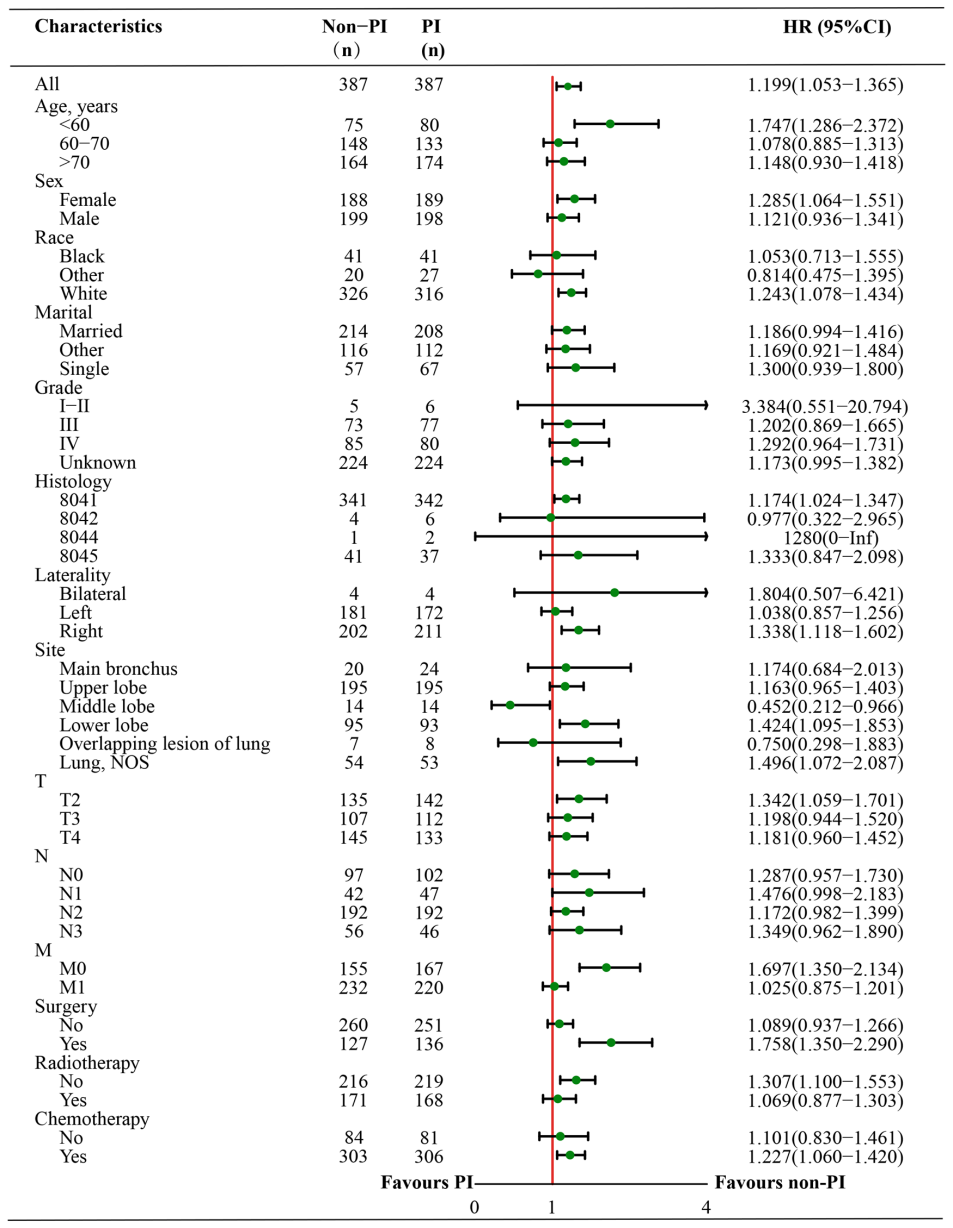
Forest plot of the subgroup analysis in SCLC patients with PI and without PI after matching.

In subgroup survival analysis, OS benefit was not observed across all subgroups in SCLC patients without PI compared with those in the PI group, except for younger age, female, white race, right laterality, lower lobe and lung, T2 stage, without distant metastasis, without radiotherapy, surgery, and chemotherapy (Figure 3).

### Baseline characteristics in the prognostic cohort

A total of 449 SCLC patients with PI who met the inclusion criteria were identified to explore the prognostic factors. For all patients with PI, the median OS time was 10 months (with a range of 1–107 months). As shown in Table 4, most of the patients were older than 60 years old. White was the majority of the population, while others counted for 17.8%. The most common T and N stage were T2 and N2, respectively. 189 patients (42.1%) underwent surgery, 180 patients (40.1%) underwent radiotherapy, and 353 patients (78.6%) underwent chemotherapy. Other variables were exhibited in Table 4. No significant difference could be found between the training and validation cohorts.

**Table 4:** Characteristics of SCLC patients with PI in the prognostic cohort.

	ALL	Training cohort	Validation cohort	*P*
	(*N* = 449)	(*N* = 314)	(*N* = 135)	
**Age**				
<60	85 (18.9%)	64 (20.4%)	21 (15.6%)	0.051
60–70	193 (43.0%)	142 (45.2%)	51 (37.8%)	
>70	171 (38.1%)	108 (34.4%)	63 (46.7%)	
**Sex**				
Female	216 (48.1%)	148 (47.1%)	68 (50.4%)	0.538
Male	233 (51.9%)	166 (52.9%)	67 (49.6%)	
**Race**				
Black	48 (10.7%)	34 (10.8%)	14 (10.4%)	0.979
Other	32 (7.1%)	22 (7.0%)	10 (7.4%)	
White	369 (82.2%)	258 (82.2%)	111 (82.2%)	
**Marital**				
Married	247 (55.0%)	169 (53.8%)	78 (57.8%)	0.110
Other	131 (29.2%)	88 (28.0%)	43 (31.9%)	
Single	71 (15.8%)	57 (18.2%)	14 (10.4%)	
**Grade**				
I–II	6 (1.3%)	4 (1.3%)	2 (1.5%)	0.825
III	103 (22.9%)	72 (22.9%)	31 (23.0%)	
IV	96 (21.4%)	64 (20.4%)	32 (23.7%)	
Unknown	244 (54.3%)	174 (55.4%)	70 (51.9%)	
**Histology**				
8041	387 (86.2%)	268 (85.4%)	119 (88.1%)	0.411
8042	6 (1.3%)	6 (1.9%)	0 (0%)	
8044	2 (0.4%)	2 (0.6%)	0 (0%)	
8045	54 (12.0%)	38 (12.1%)	16 (11.9%)	
**Laterality**				
Bilateral	6 (1.3%)	4 (1.3%)	2 (1.5%)	0.920
Left	206 (45.9%)	146 (46.5%)	60 (44.4%)	
Right	237 (52.8%)	164 (52.2%)	73 (54.1%)	
**Site**				
Main bronchus	24 (5.3%)	15 (4.8%)	9 (6.7%)	0.131
Upper lobe, lung	229 (51.0%)	162 (51.6%)	67 (49.6%)	
Middle lobe, lung	14 (3.1%)	6 (1.9%)	8 (5.9%)	
Lower lobe, lung	113 (25.2%)	86 (27.4%)	27 (20.0%)	
Overlapping lesion	10 (2.2%)	7 (2.2%)	3 (2.2%)	
Lung, NOS	59 (13.1%)	38 (12.1%)	21 (15.6%)	
**T**				
T2	174 (38.8%)	111 (35.4%)	63 (46.7%)	0.071
T3	130 (29.0%)	98 (31.2%)	32 (23.7%)	
T4	145 (32.3%)	105 (33.4%)	40 (29.6%)	
**N**				
N0	133 (29.6%)	84 (26.8%)	49 (36.3%)	0.225
N1	54 (12.0%)	41 (13.1%)	13 (9.6%)	
N2	211 (47.0%)	153 (48.7%)	58 (43.0%)	
N3	51 (11.4%)	36 (11.5%)	15 (11.1%)	
**M**				
M0	202 (45.0%)	139 (44.3%)	63 (46.7%)	0.679
M1	247 (55.0%)	175 (55.7%)	72 (53.3%)	
**Surgery**				
No	260 (57.9%)	187 (59.6%)	73 (54.1%)	0.298
Yes	189 (42.1%)	127 (40.4%)	62 (45.9%)	
**Radiation**				
No	269 (59.9%)	191 (60.8%)	78 (57.8%)	0.600
Yes	180 (40.1%)	123 (39.2%)	57 (42.2%)	
**Chemotherapy**				
No	96 (21.4%)	65 (20.7%)	31 (23.0%)	0.616
Yes	353 (78.6%)	249 (79.3%)	104 (77.0%)	

8041: small cell carcinoma, NOS; 8042: oat cell carcinoma; 8043: small cell carcinoma, intermediate cell; 8045: combined small cell carcinoma; PI: pleural invasion.

### Prognostic factors for SCLC patients with Pi

As shown in Table 5, age, tumor site, T stage, N stage, M stage, surgery, radiotherapy, and chemotherapy were identified as PI-related risk factors by univariate cox regression analysis. Then, multivariate cox regression analysis further confirmed that higher age, higher N stage, higher M stage, surgery, radiotherapy, and chemotherapy were the independent prognostic factors to predict OS in SCLC patients with PI.

**Table 5:** Univariate and multivariate Cox regression analyses of prognostic factors correlated with OS in SCLC patients with PI.

	Univariate analysis			Multivariate analysis		
	HR	95%CI	*P*	HR	95%CI	*P*
**Age, years**						
<60	Reference					
60–70	1.055	0.762–1.459	0.748	1.072	0.767–1.497	0.685
>70	1.426	1.019–1.996	0.039	1.547	1.084–2.208	0.016
**Sex**						
Female	Reference					
Male	1.055	0.830–1.343	0.661			
**Race**						
Black	Reference					
Other	0.990	0.548–1.789	0.973			
White	1.139	0.758–1.710	0.531			
**Marital**						
Married	Reference					
Other	1.141	0.867–1.503	0.346			
Single	1.142	0.822–1.587	0.430			
**Grade**						
I–II	Reference					
III	0.721	0.260–1.999	0.529			
IV	0.897	0.324–2.486	0.835			
Unknown	1.424	0.528–3.844	0.485			
**Histology**						
8041	Reference					
8042	1.140	0.506–2.566	0.753			
8044	0.769	0.191–3.098	0.712			
8045	0.697	0.473–1.027	0.068			
**Laterality**						
Bilateral	Reference					
Left	0.402	0.148–1.089	0.073			
Right	0.395	0.146–1.071	0.068			
**Tumor site**						
Main bronchus	Reference					
Upper lobe	0.533	0.306–0.928	0.026	1.021	0.574–1.817	0.943
Middle lobe	0.159	0.036–0.700	0.015	0.321	0.071–1.458	0.141
Lower lobe	0.694	0.391–1.23	0.211	1.420	0.771–2.617	0.260
Overlapping lesion	1.481	0.569–3.86	0.421	2.217	0.828–5.934	0.113
Lung, NOS	1.177	0.635–2.18	0.605	1.285	0.678–2.438	0.442
**T**						
T2	Reference					
T3	2.071	1.528–2.808	<0.001	1.300	0.923–1.830	0.133
T4	2.225	1.646–3.007	<0.001	1.077	0.746–1.554	0.692
**N**						
N0	Reference					
N1	1.468	0.972–2.219	0.068	1.699	1.101–2.621	0.017
N2	2.204	1.615–3.008	<0.001	1.794	1.220–2.637	0.003
N3	3.115	2.030–4.781	<0.001	2.218	1.324–3.718	0.003
**M**						
M0	Reference					
M1	2.735	2.116–3.535	<0.001	1.885	1.322–2.689	<0.001
**Surgery**						
No	Reference					
Yes	0.407	0.314–0.527	<0.001	0.634	0.443–0.906	0.012
**Radiotherapy**						
No	Reference					
Yes	0.703	0.549–0.902	0.006	0.654	0.495–0.864	0.003
Chemotherapy						
No	Reference					
Yes	0.719	0.533–0.969	0.030	0.527	0.377–0.735	<0.001

8041: small cell carcinoma, NOS; 8042: oat cell carcinoma; 8043: small cell carcinoma, intermediate cell; 8045: combined small cell carcinoma; PI: pleural invasion.

### Nomogram construction and validation

**Figure 4:**
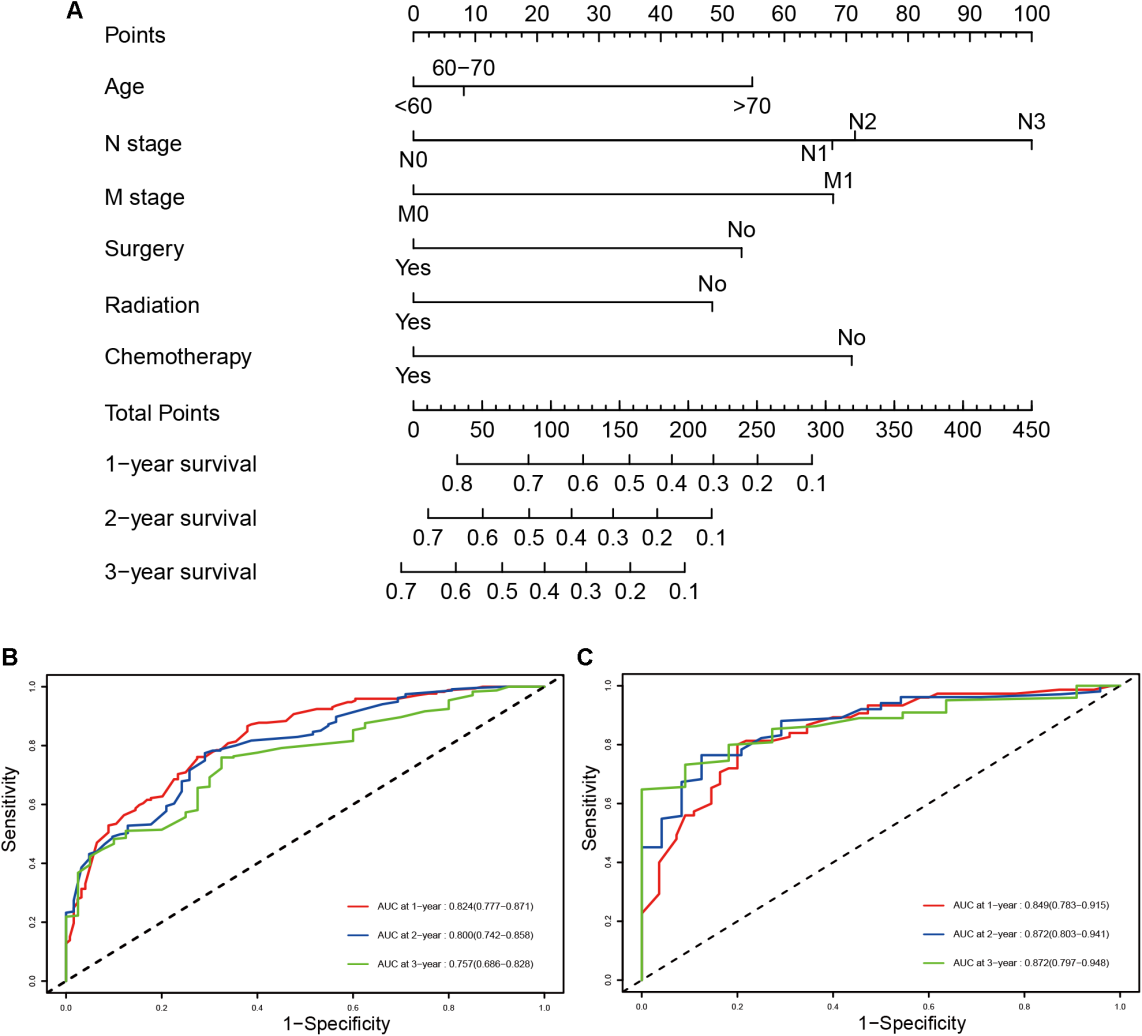
The prognostic nomogram for predicting the 1-, 2-, and 3-year overall survival for SCLC patients with PI (**A**), time-dependent ROC, and AUCs of the prognostic nomogram for1-, 2-, and 3-year overall survival in the training cohort (**B**) and validation cohort (**C**).

**Figure 5:**
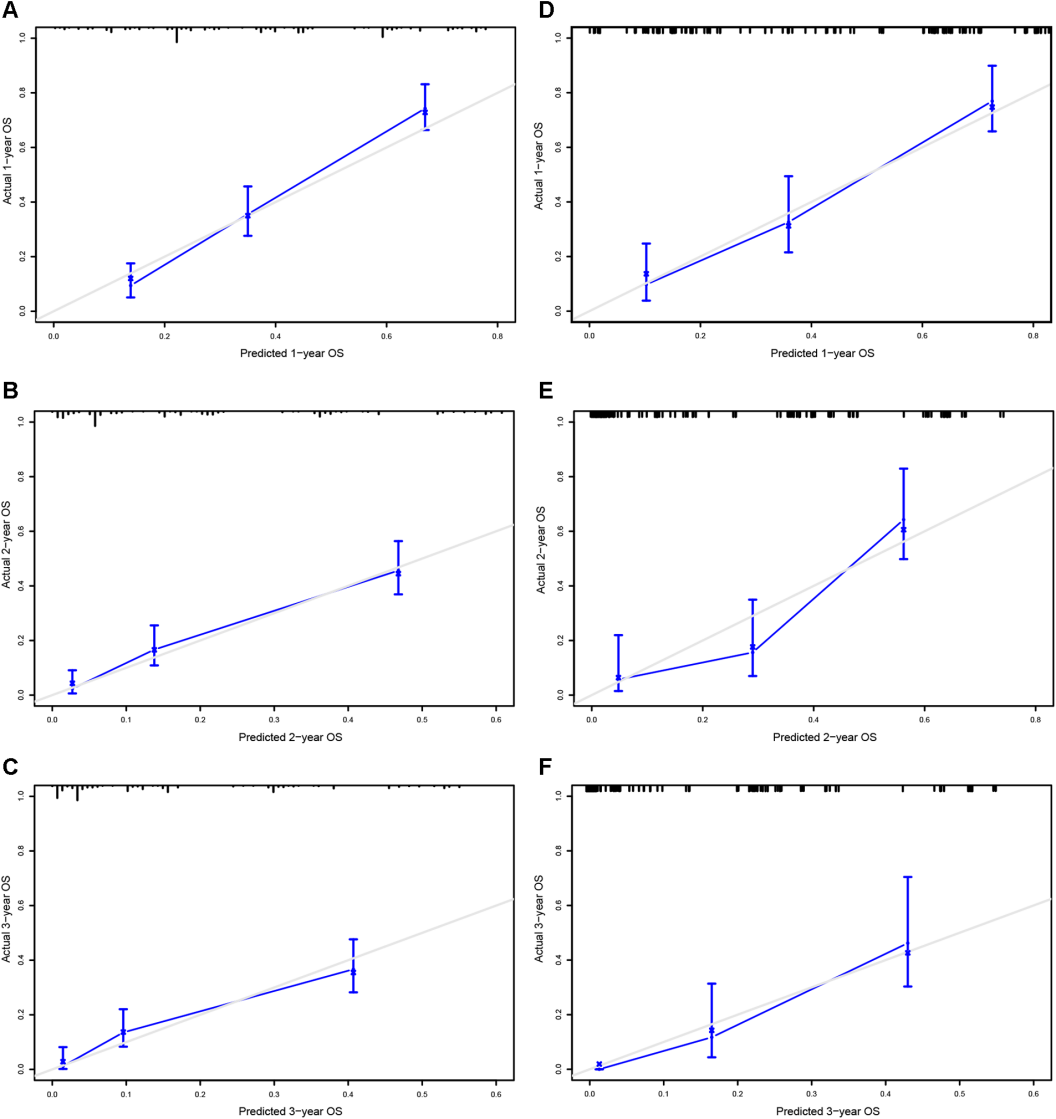
Calibrating curves of the prognostic nomogram for 1- (**A**), 2- (**C**), and 3-year (**E**) in the training cohort, while 1-(**B**), 2-(**D**), and 3-year (**F**) in the validation cohort.

**Figure 6:**
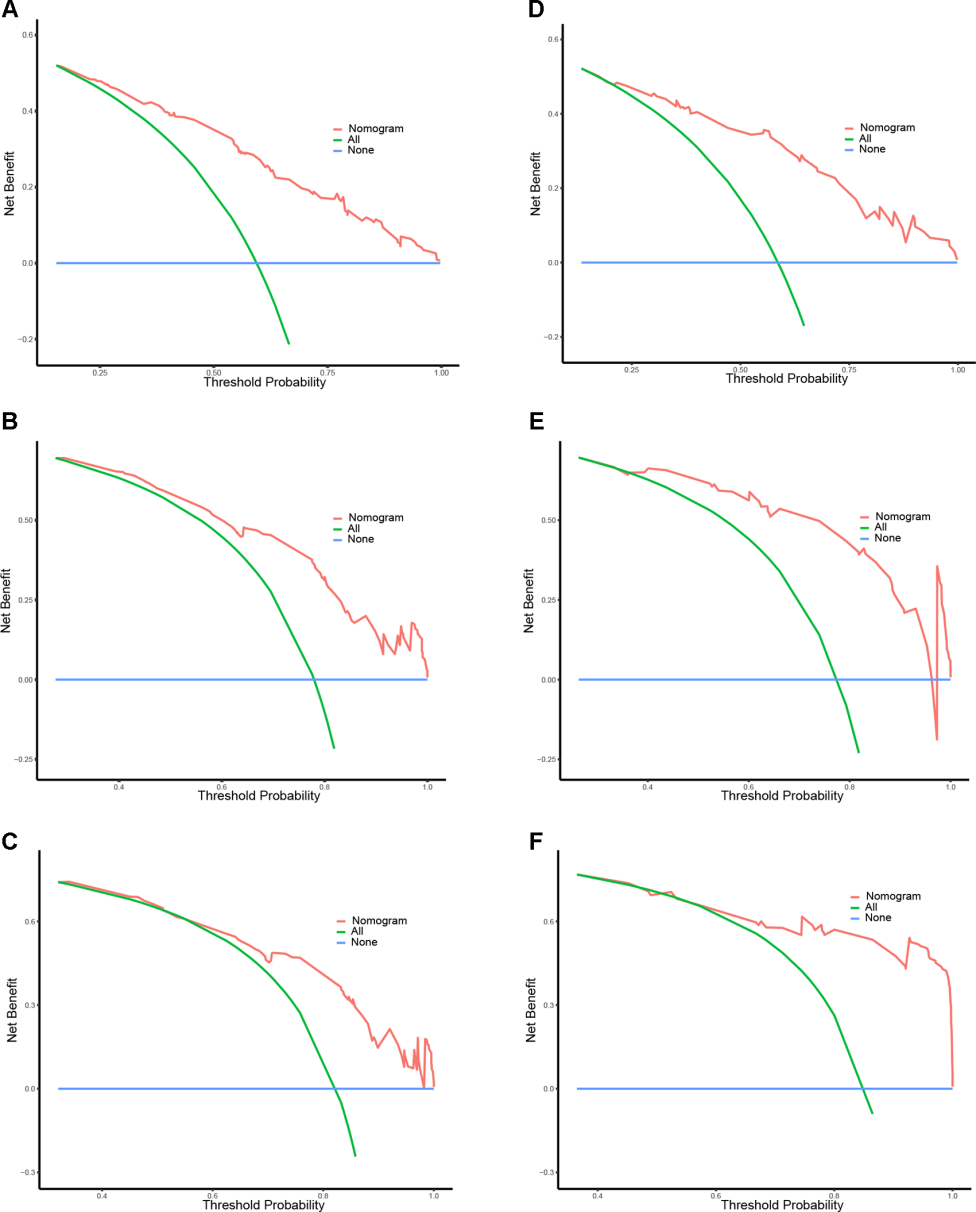
DCA curves of the prognostic nomogram for 1- (**A**), 2- (**C**), and 3-year (**E**) in the training cohort, while 1-(**B**), 2-(**D**), and 3-year (**F**) in the validation cohort.

A prognostic nomogram was established based on the six independent prognostic factors (Figure 4A). The C-index was 0.714 in the training cohort, showing a good discrimination ability of the nomogram as well as 0.746 in the validation cohort. In addition, the AUCs of the nomogram in the training cohort for the 1-, 2-, and 3-year reached 0.824, 0.800, 0.757, while 0.849, 0.872, and 0.871 in the validation cohort, respectively (Figures 4B,C). What's more, calibration curves exhibited outstanding consistency between predicted and actual overall survival at the 1-, 2-, and 3-year in the training and validation cohorts, respectively (Figure 5). DCA curves at the 1-, 2-, and 3-year indicated that the nomogram had high predictive accuracy in both cohorts (Figure 6).

## Discussion

We conducted this retrospective study based on SCLC patients with PI and without PI to investigate its prognostic value *via* the SEER database. Besides, independent prognostic factors for SCLC patients with PI were identified. By utilizing multivariate Cox regression and PSM analyses to balance the confounding factors, our results demonstrated that SCLC patients with PI were correlated with a significantly worse prognosis. Furthermore, to efficiently predict OS for SCLC patients with PI, we established a prognostic nomogram with reliable accuracy and discriminative ability which were validated by ROC, calibration, and DCA curves. This nomogram can serve as a practical tool for clinicians to identify patients with a high risk of poor survival and to determine the optimal clinical treatment for patients diagnosed with PI.

Pleural invasion was first reported in 1958 by Brewer et al. to be a poor negative survival predictor for lung cancer (18). Recently, the studies concentrated on PI about its incidence and prognostic effect are increasing gradually. The incidence of VPI is variable, accounting for approximately 11.5%–46.6% of total NSCLC cases (18–21). It is widely accepted that the presence of VPI is confirmed to be an adverse prognostic factor in NSCLC, especially in patients with early stage. In a series of 886 NSCLC patients, there was a significant difference between the patients with VPI and without VPI in the 5-year OS rates, which were 80.8%, 63.7%, and 49.6% in PL0, PL1, and PL2, respectively (22). In line with another study consisting of 1,488 patients with surgically resected non-small cell carcinoma, the OS of patients with PI was worse than those without PI and the 5-year OS rates with PL0, PL1, PL2, and PL3 tumors were 80%, 60%, 55%, and 52%, respectively (23). Other comparative studies also revealed the same poor prognosis in NSCLC patients with VPI or PI (11, 21, 24). However, there are some shortcomings in these studies. For example, some studies did not adjust for baseline confounders to assess the prognostic value of PI accurately. Other studies had a limited number of cases or were based on NSCLC. The prognostic value of PI in SCLC patients had less attention.

In the present study, we confirmed that PI was associated with statistically significantly deteriorated OS among SCLC patients in both original and matched cohorts. In addition, the current multivariate Cox regression analysis showed that PI was an independent worse prognostic factor before and after matching, consistent with previous studies showing VPI led to poor OS. Stratified analysis indicated that OS difference was not existent between the non-PI group and PI group in SCLC patients across all subgroups, except for younger age, female, white race, right laterality, lower lobe and lung, T2 stage, without distant metastasis, without radiotherapy, surgery, and chemotherapy. The number of SCLC patients reviewed who were located in the lower lobe and lung site (295/774), non-right (357/784), and non-white (129/784) was relatively low. Given the small sample size, the results cannot be accurately evaluated. Age was regarded as a prognostic factor in SCLC patients as well as our results. SCLC patients with older age may have a poor physical condition, have a high risk of metastasis, and are more likely to die from other diseases. Whether in the 7th or 8th TNM classification, PI was included in the T stage. In addition, VPI was described as T2, while parietal PI was described as T3. A retrospective analysis published by Qi et al. showed that the 5-year OS of patients without VPI was significantly better than those with VPI in NSCLC with pT2 stage (25). Our results demonstrated that PI exhibited a significant impact on survival in T2 stage, whereas had no effect in T3 and T4 stage. Maybe higher T stage is often accompanied by a high risk of metastasis or more extents of invasion which impairs the effect of PI in SCLC patients. It deserves further investigation to illuminate the phenomenon. Most studies said in lung cancer patients without node lymph node metastasis, VPI led to a worse survival than those without VPI, while no significant difference existed in patients with lymph node metastasis (12, 21). Though our study showed that the OS of patients with PI and without PI was the same regardless of lymph node metastasis, the OS seemed to favored non-PI. Perhaps, a relatively small number of patients underestimated the impact of PI in N0 diseases and N^+^ diseases. Besides, we didn't distinguish the degree of PI so their actual effect may be confused. Generally, the incidence of distant metastasis at the time of initial diagnosis of SCLC is more than 60% and overall survival and median survival are worse in patients with metastatic SCLC (26). Therefore, these may be the reason why the impact of PI in metastatic SCLC patients was not obvious in this study.

The reason for the worse prognosis caused by PI is still unclear. Perhaps, one possible reason is a high probability of lymph node metastasis in patients with PI. Because of the abundance in lymphatic vessels of the pleura, lung cancer cells in the subpleural tend to invade the pleural layer rapidly through the flow of pleural effusions in the pleural cavity. Once lymph nodes have been involved, malignant cells will further migrate by invading mediastinal lymphatic vessels (27). Like previous studies reported, VPI was significantly correlated with more extensive hilar or mediastinal lymph node metastasis (22, 28). Thus, PI may be the first step for further distant metastasis through minimal hematogenous dissemination, regardless of lymph node involvement.

Nomograms can predict prognosis efficiently in other tumors. Tang et al. combined age, sex, grade at diagnosis, number of metastatic organs, histology, and chemotherapy to predict cancer-specific survival in metastatic esophageal cancer (29). In metastatic cutaneous melanoma, a nomogram was established based on age, sex, race, marital status, insurance, number of metastatic organs, T stage, N stage, surgery, and chemotherapy (30). To date, there are many studies on the prognosis and nomograms of SCLC, nonetheless, there is still no study concentrated on the prognosis in SCLC patients with PI based on the clinical characteristics, which leads to a worse cancer prognosis. Furthermore, a novel prognostic nomogram was constructed in this study.

In a retrospective study of 1,374 NSCLC patients with stage pT1-2N2M0, Zhang et al. exhibited that the presence of VPI is a poor prognostic factor, even in patients III/N2 NSCLC (31). And beyond that, they also revealed that the OS could be improved significantly by chemotherapy, especially in non-VPI patients. What's more, Wang et al. constructed a nomogram based on age, gender, race, histology, T stage, N stage, and M stage to predict survival in NSCLC patients with VPI (27). As well as in SCLC, VPI was an indicator of a poor prognosis for SCLC with surgery, especially in those N0 diseases, besides, age, N stage, and chemotherapy were recognized as independent prognostic factors in patients with VPI (32). Although chemotherapy plus immune checkpoint inhibitors are recommended for patients with extensive-stage SCLC, platinum plus etoposide has been recommended for many years and is a common treatment of advanced SCLC in practice (33, 34). In line with these reports, age, N stage, and chemotherapy were associated with OS in our study. Distant metastasis was a strong independent factor for OS and was incorporated into the prognostic nomogram (35, 36), consistent with the nomogram in our study. In a propensity score-matched analysis of SCLC patients from America and China, surgical resection could significantly improve overall survival and lung cancer-specific survival of stage III SCLC patients (37). In another propensity-score analysis of early-stage patients with SCLC, Wang et al. demonstrated a statistically significant benefit for surgery (38). But, considering the limited number of patients with surgery in the present study, it should be cautious to make the decision on surgery in SCLC patients with PI based on the patients' conditions. Radiotherapy has been demonstrated to improve median overall survival in early stage of SCLC (39, 40), as well as in patients with PI in our study. Further investigation on benefits of radiotherapy in SCLC patients with PI is required.

However, there are still some limitations that need to pay attention in this study. First, prospective randomized controlled studies are required to confirm our results because of the selection bias of the retrospective study. Second, due to the lack of external validation in the present study, an inherent bias can not avoid. Third, because of high mortality and low survival, the small number of SCLC patients with pleural invasion may have contributed to the possible error. Besides, it may reduce the accuracy of the nomogram without histological data in SCLC with PI. Finally, the data on tumor grade and the specific degree of pleural invasion was incomplete which may underestimate their impact on overall survival. The survival effect of specific degree of tumor grade and pleural invasion should be further investigate in the future.

## Conclusions

Our study demonstrated that the existence of PI in SCLC patients had a statistically significant adverse impact on survival. At the same time, we comprehensively demonstrated that age, N stage, M stage, surgery, radiotherapy, and chemotherapy were the independent prognostic factors for SLCLC patients with PI. The nomogram may beapplied as a clinically useful to assess the prognosis of SCLC patients with PI and could facilitate clinical decision-making.

## Data Availability

The datasets presented in this study can be found in online repositories. The names of the repository/repositories and accession number(s) can be found below: http://www.seer.cancer.gov/.
